# Optimizing blood pressure control by an Information Communication Technology-supported case management (PIA study): study protocol for a cluster-randomized controlled trial of a delegation model for general practices

**DOI:** 10.1186/s13063-021-05660-4

**Published:** 2021-10-25

**Authors:** Arian Karimzadeh, Frauke Leupold, Anika Thielmann, Nicola Amarell, Kerstin Klidis, Verena Schroeder, Christine Kersting, Claudia Ose, Karl-Heinz Joeckel, Birgitta Weltermann

**Affiliations:** 1 Institute of Family Medicine and General Practice, Medical Faculty of the University of Bonn, Venusberg-Campus 1, 53127 Bonn, Germany; 2 Center for Clinical Trials, University Hospital Essen, University of Duisburg-Essen, Hufelandstr. 55, 45147 Essen, Germany; 3 Institute for General Medicine, University Hospital Essen, University of Duisburg-Essen, Hufelandstr. 55, 45122 Essen, Germany; 4 Institute for Medical Informatics, Biometry and Epidemiology, University Hospital Essen, University of Duisburg-Essen, Hufelandstr. 55, 45147 Essen, Germany

**Keywords:** Hypertension, Blood pressure, Telemedicine, Family medicine, General practice, Home blood pressure monitoring, Delegation, mHealth, Information technology, Mobile application

## Abstract

**Background:**

Longitudinal hypertension control prevents heart attacks, strokes, and other cardiovascular diseases. However, 49% of patients in German family medicine practices do not reach blood pressure (BP) targets (< 140/90 mmHg). Drawing on successful international approaches, the PIA study introduces the PIA information and communication technology system (PIA-ICT) for hypertension management in primary care. The PIA-ICT comprises the PIA-App for patients and the PIA practice management center for practices. Case management includes electronic communication with patients, recall, and stepwise medication adjustments following guidelines. The system supports a physician-supervised delegation model to practice assistants. General practitioners are qualified by eLearning. Patients learn how to obtain reliable BP readings, which they communicate to the practice using the PIA-App.

**Methods:**

The effectiveness of the PIA-Intervention is evaluated in a cluster-randomized study with 60 practices, 120 practice assistants, and 1020 patients. Patients in the intervention group receive the PIA-Intervention; the control group receives usual care. The primary outcome is the BP control rate (BP < 140/90 mmHg) after 12 months. Using a mixed methods approach, secondary outcomes address the acceptance on behalf of physicians, practice assistants, and patients. This includes an evaluation of the delegation model.

**Discussion:**

It is hypothesized that the PIA-Intervention will improve the quality of BP care. Perspectively, it may constitute an important health service model for primary care in Germany.

**Trial registration:**

German Clinical Trials Register DRKS00012680. Registered on May 10, 2019

**Supplementary Information:**

The online version contains supplementary material available at 10.1186/s13063-021-05660-4.

## Administrative information

**Table:** 

Title {1}	Optimizing blood pressure control by an ICT supported case management (PIA study): study protocol for a cluster-randomized controlled trial of a delegation model for general practices
Trial registration {2a and 2b}.	German Clinical Trials Register, DRKS00012680. Registered May 10th 2019, https://www.drks.de/drks_web/setLocale_EN.do.
Protocol version {3}	Protocol Version 1.0, 22.03.2021
Funding {4}	German Innovation Fund, located at the Federal Joint Committee (Innovationsausschuss beim Gemeinsamen Bundesausschuss, G-BA)
Author details {5a}	1 Institute of Family Medicine and General Practice, Medical Faculty of the University of Bonn, Venusberg-Campus 1, 53127 Bonn, Germany 2 Center for Clinical Trials, University Hospital Essen, University of Duisburg-Essen, Hufelandstr. 55, 45147 Essen, Germany. 3 Institute for General Medicine, University Hospital Essen, University of Duisburg-Essen, Hufelandstr. 55, 45122 Essen, Germany 4 Institute for Medical Informatics, Biometry and Epidemiology, University Hospital Essen, University of Duisburg-Essen, Hufelandstr. 55, 45147 Essen, Germany
Name and contact information for the trial sponsor {5b}	Institute for Family Medicine and General Practice, University of Bonn; Prof. Dr. Birgitta Weltermann, birgitta.weltermann@ukbonn.de
Role of sponsor {5c}	The sponsor conceptualized the trial and is responsible for the setup of the consortium, as well as the management of the study and respective publications.

## Introduction

### Background and rationale {6a}

Hypertension is a global public health problem with an estimated number of more than one billion people affected. Despite evidence-based therapeutic options available, it is a leading cause of premature death [1]. Reaching blood pressure (BP) targets of < 140/90 mmHg is associated with significant reductions in cardiovascular events [2, 3]. A meta-analysis of 47 randomized controlled trials with 153,825 patients showed that a BP reduction of 10 mmHg systolic and 5 mmHg diastolic reduces the relative risk for major outcomes after 5 years: heart failure by 43%, stroke by 36%, cardiovascular death by 18%, and coronary heart disease by 16% [3]. However, guideline-recommended BP targets are not reached by 49% of family medicine practice patients in Germany [4]. A variety of well-documented factors play a role, e.g., insufficient adherence to diagnostic and therapeutic algorithms by physicians, poor medication adherence by patients, lack of organizational concepts supporting recall, and delegation to non-physician staff [5–8].

A Cochrane review of various interventions showed the best effects on hypertension control if strategies targeting patients, physicians, and organizations are combined [8]. Recently, complex interventions integrating information and communication technologies (ICT) and delegation to non-physician personnel were successful [9]. Margolis et al. [9] developed an ICT-supported case management involving a delegation model to pharmacists: patients transmitted BP self-monitoring results electronically to a clinical pharmacologist who adjusted drug regimes. After 12 months, intervention effects of − 9.7/− 5.1 mmHg systolic/diastolic were observed; the BP control rate in the intervention group was 18% higher than that in the control group (71% vs 53%) [9]. A meta-analysis of 33 studies on hypertension management delegated to non-physician staff (nurses) showed better BP reductions than standard care (systolic − 8.2 mmHg) [10]. Interventions with nurses who were allowed to prescribe and adjust medications achieved effects of − 8.9/− 4.0 mmHg [10]. Similar results were achieved in a physician-guided, nurse-managed hypertension management which used patient self-measurements and drug algorithms: after only 6 months, an intervention effect of − 8.5/− 3.1 mmHg was observed [11]. This effect was achieved by four times more frequent drug adjustments in the intervention group compared to the control with standard care (*p* < 0.01).

Based on these results, the PIA-Intervention was designed as an ICT-supported case management for the German general practice setting: the PIA-Intervention allows for a highly secured, electronic communication between patients (PIA-App for smartphone/tablet) and practices (PIA practice management center, PIA-PrMC). Patients learn to obtain and transmit reliable BP readings to the practice using the PIA-App; trained practice personnel provide electronic feedback with adjusted medication plans. The concept includes a physician-supervised delegation to practice assistants who manage recall, electronic communication with patients, and step-wise medication adjustments under physician supervision.

### Objectives {7}

The main study objective is to investigate if the PIA-Intervention improves BP control rate (BP ≤ 140/90 mmHg) after 12 months in patients with uncontrolled hypertension at baseline.

The PIA-Intervention comprises the following:

1. The PIA-ICT (PIA-App for patients and PIA-PrMC for practices) for patient-physician communication, recall, and step-wise medication adjustments
2. eLearning for general practitioners and practice assistants
3. Patient education on valid BP readings by practice staff and access to information on hypertension by PIA-App

The concept realizes a physician-supervised delegation model for hypertension management.

### Trial design {8}

The study is designed as a prospective cluster randomized controlled trial (cCRT) with an intervention and a waiting list control group. A 1:1 randomization takes place at the practice level, i.e., all patients of a practice are assigned to either the intervention or the control group (30 practices per study arm). The cluster approach is chosen to avoid contamination between the intervention and control groups.

**Fig. 1:**
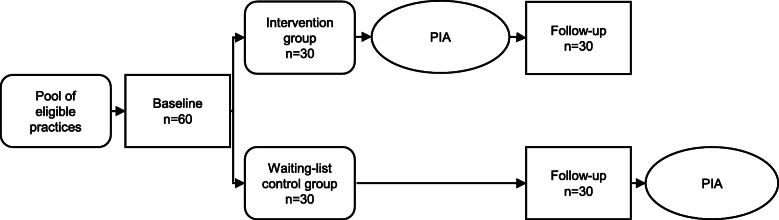
Study design: a cluster-randomized controlled trial with an intervention and a waiting list control group

While the control group receives standard care, the intervention group will use the PIA-Intervention for 12 months. After collection of the follow-up data, the control group will receive access to the PIA-ICT for 3 months (waiting list control). The framework is a superiority approach. For details, see Fig. 1.

## Methods: participants, interventions, and outcomes

### Study setting {9}

The study is conducted in German general practices with certified general practitioners (GP) who are eligible to serve patients insured in the statutory health insurance.

### Eligibility criteria {10}

#### Eligibility criteria on practice level

All of the following inclusion criteria apply: (a) certified GP eligible to serve patients insured in the statuary health insurance, (b) practice is equipped with at least one practice computer with Internet access (Windows 7 or higher), and (c) participation of at least one GP and up to three practice assistants per practice.

The exclusion criteria are as follows: (a) GP has an additional qualification in hypertensiology and/or (b) participated in the development of the intervention.

#### Eligibility criteria on patient level

All of the following inclusion criteria apply: (a) age 40 to 79 years, (b) diagnosed with essential hypertension (ICD I10), (c) resting practice BP > 140/90 mmHg (calculated as the mean value of the 2nd and 3rd BP readings obtained by trained personnel), (d) need or use at least ≥ 1 antihypertensive substance (drug), (e) insured by the statutory health insurance, (f) equipped with smart devices (tablet or smartphone with android 6 or higher), (g) sufficient skills to use the smartphone or tablet (defined as device use at least 3 times a week), and (h) has sufficient language skills to understand the study documents.

The exclusion criteria are as follows: (a) known white coat hypertension, (b) critical health conditions at the time of inclusion (e.g., hypertensive crisis, BP-related symptoms such as dizziness or headache), (c) chronic renal failure requiring dialysis, (d) being pregnant or breastfeeding, (e) hyperkalemia, (f) secondary hypertension (e.g., renal artery stenosis), and (g) heart failure NYHA III or IV.

### Who will take informed consent? {26a}

The research team will obtain written informed consent from all participating practice owners, employed physicians, and practice assistants. The physicians and/or practice assistants obtain written informed consent from all patients during practice visits.

### Additional consent provisions for collection and use of participant data and biological specimens {26b}

Not applicable.

### Interventions

#### Explanation for the choice of comparators {6b}

The PIA-Intervention is compared to usual care. Usual care is the standard comparator for ICT-based interventions for hypertension management [12], including those implementing a delegation model to non-physician staff [10].

#### Intervention description {11a}

**Fig. 2:**
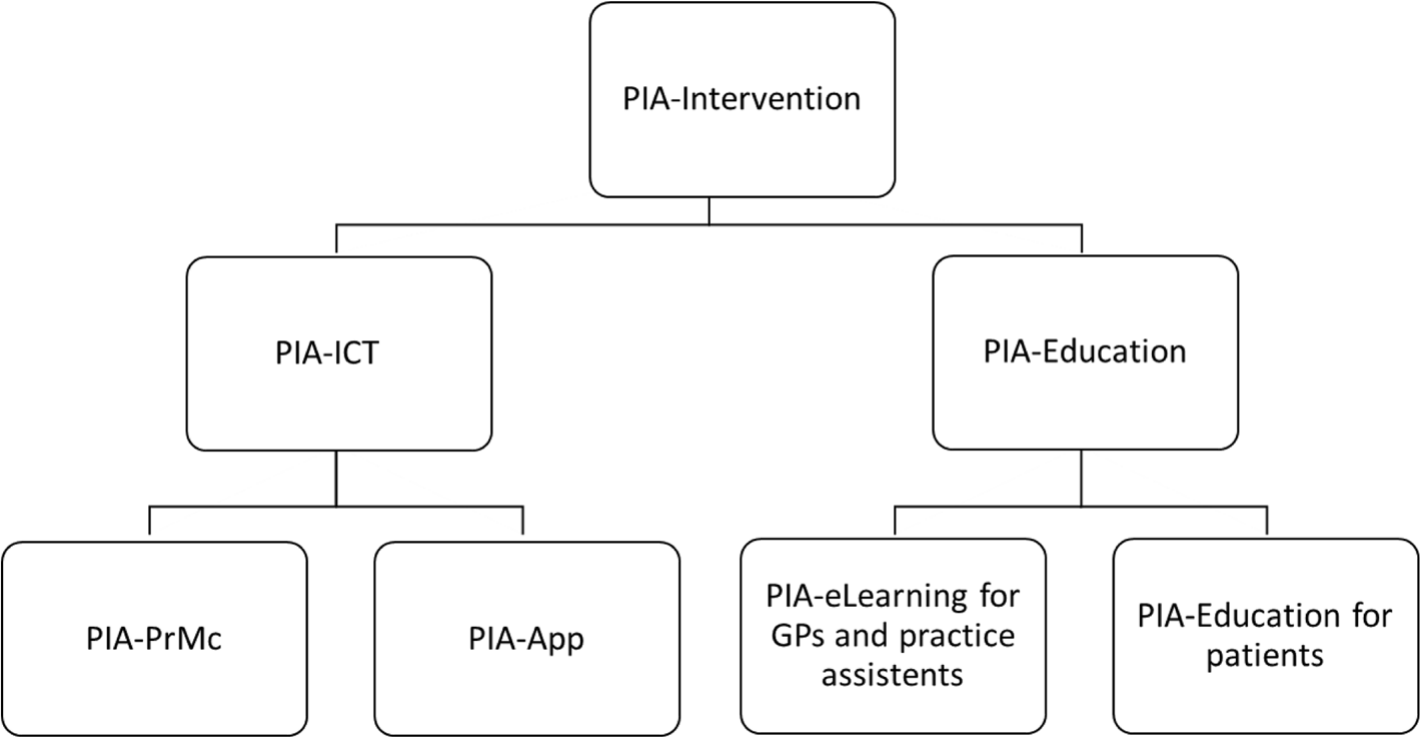
The elements of the PIA-Intervention

As a complex intervention, the PIA approach to improve hypertension management comprises the PIA-ICT (PIA-App and PIA-PrMC) and PIA-Education (eLearning/on-site trainings for practice teams and patients) with four elements (for details, see Fig. 2 and Table 1).

**Table 1:** The PIA-Intervention: elements and target groups

	PIA-ICT		PIA-Education		
	PIA-Practice management center (PIA-PrMC)	PIA-App	PIA-eLearning and on-site teaching		PIA-Patient-Education
Setting	Secured system in practice	On patients’ smartphone/tablet	PIA-Website for practices (secured login) and on-site teaching		In practice and PIA-App
Target croup	GPs and practice assistants	Patients	GPs	Practice assistants	Patients
Functions/content	1. Secured patient-practice communication 2. Recall and step-wise medication adjustments 3. Communication GP and practice assistants 4. Pre-defined algorithms for medication regimes 5. Graphic display of BP data and target range over time 6. Electronic transmission of medication plan, physician PIN required 7. Data transfer to the trial center 8. Data download for the practice	1. Secured patient-practice communication 2. Transfer of BP values 3. Display of current medication plan 4. BP history displayed as a graph for different time grids 5. Request for prescription refill	1. Evidence-based information on hypertension 2. How to use the PIA-PrMC 3. Details of the delegation model 4. Information on the study processes	1. Evidence-based information on hypertension including medication classes 2. How to use the PIA-PrMC 3. How to use the PIA-App 4. How to obtain valid BP measurements in practice and at home 5. Information about the study processes	1. How to use the PIA-App 2. How to obtain valid BP measurements (in practice training and video) 3. Access to websites with evidence-based information on hypertension

The concept realizes a physician-supervised delegation model for hypertension management. The PIA-ICT was conceptualized by researchers of the Institute of Family Medicine and General Practice of the University of Bonn. It was realized together with experts for medical informatics from a private company following an agile design. The software solution was piloted in primary care practices with GPs, practice assistants and patients prior to its use in this cCRT.

1. PIA-App for patients (PIA-App): The PIA-App is a patient-facing application for smart devices (smartphone or tablet with Android operating system) that allows a secured communication between patient and practice. The key communication feature is the transmission of BP readings from the patient to the practice and the transfer of adjusted medication plans from the practice to the patient, paralleled by short text messages in both directions. The patient receives a push notification when the practice has sent a new medication plan. To motivate patients’ adherence, a graph displays BP data over time including systolic BP (SBP), diastolic BP (DBP), pulse rate, and the BP target range. The BP target is set at < 135/85 mmHg per standard but can be individualized by the GP. In addition, the PIA-App enables patients to request prescription refills and access to a video on how to obtain a valid BP measurement as well as web links related to hypertension.
2. PIA practice management center (PIA-PrMC): The PIA-PrMC is a Windows application, which is used by GP and practice assistants to manage patients’ hypertension therapy. It allows for reviewing patients’ BP records and to adjust drug regimes. Integrated into the application, processes realize a delegation model: First, the GP enters his individual preferences regarding medication regimes. These can be individualized as needed for each patient. Second, the practice assistant reviews the incoming BP readings and—as long as target BP values are not reached and medication is tolerated—adjusts the pre-set medication regime and writes a suggestion for a short note to the patient. Third, the GP reviews these suggestions and, if approved, initiates the electronic transmission to the patient’s PIA-App using a physician personalized identification number (PIN). If a GP does not agree with the practice assistants’ suggestions and prefers a different medication regime or text message, the physician communicates this to the practice assistant who executes these physician orders with identical subsequent procedures. Fourth, the PIA-PrMC supports the electronic recall of patients who did not transmit BP readings or have questions regarding their BP care.
  The medication options offered to GPs follow the current hypertension guidelines as issued by the European Society of Hypertension [2]. For convenience, pharmaceutical agents are displayed according to the regional prevalence of their use in previous years. For each pharmaceutical agent, a typical regime for step-up dosing is integrated, thus allowing for an advanced treatment plan for each patient. In case of adverse drug reactions or other needs to adjust medication choices, the patient’s medication plan can be adjusted individually by the GP.
  Data management for the study is facilitated by an export function of aggregated, pseudonymized data in a data file format.
3. PIA-eLearning for GPs and practice assistants:

- *PIA-eLearning for physicians*: An audio-visual learning video with about 35 slides introduces GPs to the PIA-Intervention: how to use the software and the delegation concept with the new role for practice assistants (PIA practice assistant) within the German legal frame. The latter allows for the delegation of specific tasks to practice assistants and nurses, but not substitution, i.e., no issuing of prescriptions and no independent medication adjustments.
- *PIA-eLearning and PIA certificate to qualify practice assistants (PIA practice assistant)*: Audio-visual learning videos with about 50 slides and a video on BP self-measurements trains practice assistants on the following topics: how to measure BP according to standard, hypertension as so-called silent killer, sequelae of untreated hypertension, BP targets, drug regimens according to guidelines, how to implement the medication step-up based on physician-defined algorithms, short text message communication via the PIA-PrMC, patient management, and recall using the PIA-PrMC. In addition, practice assistants learn how to conduct the study at their practice site and how to educate patients regarding home BP readings. After the eLearning, practice assistants take a written examination and, if passed, receive the PIA certificate. eLearning material will be provided for download via a secured web space.

4. PIA in-practice and in-app education of patients: After informed consent and study inclusion, the PIA practice assistant will teach each patient individually how to obtain valid BP readings and how to use the PIA-App including the in-app video on valid BP home measurements and further web links.

#### Criteria for discontinuing or modifying allocated interventions {11b}

Patients unable to use the PIA-App for any reason despite having received appropriate training (e.g., worsening overall health status, admission to nursing home, participant withdrawal) will discontinue the trial. The reasons will be recorded and analyzed.

#### Strategies to improve adherence to interventions {11c}

Patients in the intervention group will receive an upper arm electronic BP measuring device (BOSO family 4) for use in the study that they may keep afterwards. The PIA practice assistants will analyze the patients’ submitted BP readings at least once a week and will provide appropriate feedback under supervision. Patients not answering to electronic reminders will be contacted by phone if no BP data are transmitted for several weeks. Practices in both study arms will receive financial reimbursement per participating patient.

#### Relevant concomitant care permitted or prohibited during the trial {11d}

Not applicable.

#### Provisions for post-trial care {30}

A study insurance is covering all study-related adverse patient outcomes.

### Outcomes {12}

The following primary and secondary outcomes will be analyzed for each patient, both study groups (intervention, control) and the total population, if appropriate.

#### Primary outcome

The primary outcome is the BP control rate (% of patients with controlled BP). The BP is defined as “controlled” if the practice BP reading is in the target range, i.e., ≤ 140/90 mmHg (calculated as the mean value of the 2nd and 3rd BP readings, 3 successive measurements at intervals of 1 min each in a seated position).

The selected outcome “BP control” is internationally recognized as a surrogate parameter for the prevention of secondary diseases [13]. A meta-analysis of 47 randomized controlled trials with 153,825 patients showed that a BP reduction of 10 mmHg systolic and/or 5 mmHg diastolic reduces the relative risk for major outcomes after 5 years: heart failure by 43%, stroke by 36%, cardiovascular death by 18%, and coronary heart disease by 16% [3].

#### Secondary outcomes

The following are the secondary outcomes: (1) changes of SBP and DBP practice measurements per patient, (2) medication use and changes over time (number, kind, and dosing of antihypertensive medications used), (3) frequencies and kind of cardiovascular events (myocardial infarction, stroke, other) or death within the study period, (4) number of hospitalizations (emergency room treatments and/or in-hospital stays) and their causes care, (5) quality of life, (6) patients’ satisfaction regarding hypertension treatment by GP practice, (7) time to BP control, (8) patients’ health literacy, (9) medication adherence, (10) perceived time to invest for hypertension management in general (by physicians, practice assistants, and patients) (e.g., estimated duration of office consultation including waiting time, BP measurements, and prescription refills), (11) number of physician consultations and practice visits, and (12) perceived workload of the GPs and practice assistants by hypertension management and, for the intervention group only, (13) number of contacts between the practice assistant and patients via PIA-ICT including use of safety functions if applicable and (14) satisfaction and acceptance of PIA-Intervention by patients, GPs, and practice assistants. For details on time points, see Additional File 1: Table S2.

The following secondary data will be provided for patients insured in the respective statutory health insurance, which is a consortium partner: (1) emergency room treatments, (2) hospitalizations (e.g., in-hospital days), (3) prescription details, and (4) death (date and cause of death).

#### Participant timeline {13}

The schedule of enrollment, intervention, and assessments is shown in Additional File 1: Table S2.

#### Sample size {14}

The sample size was calculated based on the primary outcome. Using data from the study of Margolis et al. [9], we assumed a BP control rate of 65% in the intervention group and 50% in the control group after 12 months. The sample size calculation was performed in PASS V14, using an unpooled 2-sided *Z*-test to compare two proportions in a cluster-randomized design. Assuming that both study arms comprise the same number of clusters (practices), the inter-cluster coefficient is 0.055 [14] and the mean cluster size is 15 patients, 2 × 405 = 810 patients (27 clusters per study arm) are required to detect a group difference of 15% (65% vs 50%) with a power of 90%. Although the case number calculation is based on a 2-sided *Z*-test with an unpooled variance in a cluster-randomized setting, there is sufficient power for the calculation of generalized linear mixed models (GLMM) with additional covariates. Assuming a case number of 810 patients and incidence rates of cardiovascular events between 2 and 5% [3], it is a probability of 100% to observe at least one such event (PASS V14). Based on experience from a previous CRT on hypertension management by GP [15], a 10% drop-out rate in the practices is assumed; therefore, 3 more practices are recruited per study arm. Therefore, the study aims for a cluster-randomized trial with 60 GP practices (30 intervention, 30 control) with one physician, two practice assistants, and 17 patients each (total 1020 patients).

##### Changes required due to the SARS-CoV-2 pandemic

The scheduled recruitment period was influenced by the pandemic, which led to more SARS-CoV-2-related workloads and perceived insecurities on behalf of practices as well as fewer practice visits by patients due to social distancing and lockdown regulations. Accounting for this unexpected interference, the scheduled recruitment period for both practices and patients needed to be prolonged. To support recruitment, statutory health insurances involved in the project applied several patient information strategies. In addition, given an overall limited project time, the target number of patients was reduced based on a re-calculation of the sample size by the evaluator assuming a power reduction from 0.9 to 0.8 (with all other parameters kept identical) resulting in a target patient number of 2 × 300 = 600 patients (20 clusters per study arm) to detect a group difference of 15% (65% vs 50%). Assuming a 10% drop-out rate for practices and a 10% dropout rate for patients, three more practices and 2 more patients in each practice will be recruited. In summary, this cluster-randomized trial will be conducted with 46 GP practices (23 intervention, 23 control) with one GP, two practice assistants, and a mean of 17 patients each (total 782 patients). In addition, the minimum duration of the intervention was reduced to at least 6 months. This is justified by international studies of ICT delegation models which showed significant improvements in hypertension control already after this shorter period [9, 16, 17].

#### Recruitment {15}

##### Recruitment of practices

Recruitment follows a multi-stage procedure. Based on the contact data available, practices are invited by mail, fax, and/or email. Invitation materials include the study information and the consent form for participating GPs. Subsequently, practices are contacted by phone. After written consent of the participating GP, each practice is randomized. Afterwards, a clinical monitor visits each intervention and control practice to provide detailed information on the study and the study materials. Practices declining participation or not providing feedback receive a standardized non-responder questionnaire by fax for subsequent quantitative analysis.

##### Recruitment of patients

Within each practice, the recruitment of patients is coordinated by practice assistants supported by the GP as needed. To avoid selection bias, practices are requested to list all patients with a diagnosis of essential hypertension in their electronic patient management system. Practices are asked to screen all these patients regarding the inclusion criteria and, if applicable, to ask for study participation during their next routine visit. Each practice follows this approach up to the inclusion of at least 17 patients. Practices are asked to systematically document the recruitment including reasons for non-participation.

##### Changes due to the SARS-CoV-2 pandemic

As described in the “Sample size {14}” section, the pandemic interfered with the recruitment of practices and patients. Thus, a much larger number of practices needed to be contacted to recruit the target number. Also, lock-down periods in Germany from fall to spring 2020/2021 led to dropouts of recruited practices requiring additional practice recruitments. Thus, the total recruitment time had to be extended from 6 months to 12 months in total. Given large regional and inter-practice variations of pandemic burden, practices from both groups were asked to include additional patients if possible, to compensate for practices with lower patient recruitment. Nonetheless, approaches for recruitment of practices and patients remained identical over time.

### Assignment of interventions: allocation

#### Sequence generation {16a}

The randomization is conducted by the independent trial center responsible for data management and monitoring. The allocation sequence is computer-generated based on random numbers. Stratified block randomization (1:1) is used to ensure a balanced distribution of urban and rural localized practices in the intervention and control arm.

#### Concealment mechanism {16b}

For each practice recruited, the trial center will communicate the allocation in written form to the researchers of the Institute of Family Medicine and General Practice.

#### Implementation {16c}

The trial center, which is not involved in recruitment processes, generates the allocation sequence. The Institute of Family Medicine and General Practice enrolls physicians/practices. After a practice is randomized by the trial center, the institute informs the practice about the allocation. All practices enroll patients.

### Assignment of interventions: blinding

#### Who will be blinded {17a}

Blinding of involved scientists, practice personnel, and patients is not possible due to the ICT-based intervention which is offered to the intervention group only. Data analysts will follow predefined standard operating procedures for analysis to avoid bias.

#### Procedure for unblinding if needed {17b}

Not applicable.

### Data collection and management

#### Plans for assessment and collection of outcomes {18a}

Measurement instruments address patients, GPs, and practice assistants. For details on points in time, see Additional File 1: Table S2.

The following are the patients’ measurements:

1. Blood pressure measurements: All patients receive standardized practice BP measurements by trained practice assistants. For details, see the “Outcomes {12}” section. In the intervention group, only BP measurements from home BP measurements will be analyzed as transmitted electronically to the PIA-PrMC.
2. Mental well-being during the last 14 days is assessed using the WHO-Five Well-Being Index (WHO-5,1998 version, in German) [18–20]. It consists of 5 items on a 6-point Likert scale (5 = “all of the time” to 0 = “at no time”). The scores are added to a sum score ranging from 0 to 25, which is multiplied by 4 to achieve the final score with 0 denoting the worst and 100 representing the best subjective well-being [18].
3. The usability of the PIA-ICT is measured using the standardized and validated System Usability Scale (SUS) which consists of 10 questions on a 5-point Likert scale (1 = “strongly disagree” to 5 = “strongly agree”) [21, 22]. The total score ranges from 0 to 100, with a higher score indicating greater usability. An average SUS score of 70 or more is considered appropriate [22].
4. Medication adherence is measured using the standardized and validated Medication Adherence Rating Scale (MARS-D, German version) [23]. It consists of 5 items on a 5-point Likert scale (1 = “always” to 5 = “never”) which yields a sum score between 5 and 25 points with a higher score indicating better medication adherence [23].
5. Acceptance and use of the PIA-ICT are measured using the Unified Theory of Acceptance and Use of Technology model (UTAUT) which consists of 18 questions on a 5-point Likert scale (1 = “strongly disagree” to 5 = “strongly agree”). It assumes that behavioral intentions (3 items) and effective use of technology are influenced by four determinants for acceptance: performance expectancy (4 items), effort expectancy (4 items), social influence (3 items), and facilitating conditions (4 items) [24, 25].
6. Patients’ characteristics: Sociodemographic characteristics, risk factors (for example, physical activity and smoking behavior), medication adherence, management of BP self-readings, and state of general health are requested. For each patient, the GP completes a sheet addressing the patients’ medical history regarding hypertension, hypertension-related diseases, hospitalizations, other diagnoses, and details on medication and their changes during the study.

The following are the measurements addressing physicians and practice assistants:

1. Occupational self-efficacy of physicians is measured using a short version of the Occupational Self-Efficacy Scale [26, 27]. The instrument consists of 8 items on a 6-point Likert scale (6 = “totally disagree” to 1 = “totally agree”) with a higher sum score indicating a higher occupational self-efficacy.
2. Sociodemographic and professional characteristics of GPs and practice assistants: age, sex, professional degree(s), additional qualifications, number of years in practice, and working full-time or part-time.
3. Intervention group only: SUS [22] and UTAUT [24, 25] questionnaires as described above (see section 18a, patients’ measurements).
4. Given the SARS-CoV-2 pandemic, two questions were added for physicians and practice assistants addressing the perceived burden due to the pandemic.

#### Plans to promote participant retention and complete follow-up {18b}

##### Patient-directed strategy

Patients in the intervention group will be contacted by the practice via PIA-App, subsequently by phone, if no BP values are transmitted for several weeks.

##### Practice-directed strategy

Regular faxes by the institute ask for practices’ actual patient recruitment numbers and if any support is needed. The research team offers practice-specific support including practice visits regarding recruitment and use of the PIA-ICT. The institute will record all questions and support measures.

Discontinuing patients and the respective reasons are recorded by the practices; the research team records discontinuing practices and respective causes. All data available from discontinuing patients and/or practices will be analyzed.

##### Data management {19}

Data management will be carried out by the trial center according to standardized procedures as defined in the current standard operating procedures (SOPs). The data management system used by the trial center has an integrated audit trail and is Good Clinical Practice (GCP)-compliant. Data will be entered by appropriately trained data entry staff who are familiar with the study specifics. Double data entry will be used to ensure data quality for paper-based information. Data from the PIA-PrMC will be transmitted electronically to the study centers. Missing data will be addressed by imputation methods according to standard [60]. All personal data will be kept confidential in an access-restricted database. All analyses will be performed using pseudonymized data. The pseudonymized data will be stored at the ZKSE, University Hospital Essen, and the Institute of Family Medicine and General Practice, University of Bonn. The latter institute will manage the access to the data set.

##### Confidentiality {27}

Confidentiality issues and data protection issues are part of the ethics statement. The data protection agency of the University Hospital Bonn had agreed to the following approaches:

1. Confidentiality regarding patients’ and practices’ data

Contact data of practices and personnel involved are stored in access-restricted data files at the institute and the trial center. GPs’ and practice assistants’ questionnaire data will be managed as pseudonymized data files.

All personal information of patients will remain in the practices. The names of enrolled patients will be kept at the practices in a separate access-restricted paper file. The data analysis will be performed with pseudonymized data only to allow for maximum protection of participants. Information on potential participants which were not enrolled will remain solely in each practice. Before, during, and after the trial, all data will be stored in the institute and the trial center in access-restricted files according to their standard operating procedures.

2. Confidentiality in PIA-ICT (PIA-App and PIA-PrMC software)

The PIA-App will not store any personal data on the patient’s smart device. The regularly transmitted BP data does not contain any personal data. The communication between the PIA-App and the PIA-PrMC, i.e., data transfer and transmission, takes place via a secured server at the University Hospital Bonn. This communication and data transfer is encrypted by using https/Transport layer Security (TLS) with encryption algorithms on the elliptic curve and perfect forward secrecy (TLS negotiation BSI TR-021202-2). After the user (GP, practice assistant) logs on to the PIA-PrMC, a token is generated for encrypted communication with the PIA-App (Bearer Token). This token is transmitted with every communication.

3. Data transfer to trial center and the institute

Pseudonymized patient and practice data will be exported from the PIA-PrMC. This data is first stored on the GP’s practice computer. Exports will only contain pseudonymized data, i.e., personal data such as surname, name, and date of birth of the patients are removed prior to export. The export is a zip file with AES (Advanced Encryption Standard) password encryption. This file is transmitted electronically to the Institute for Family Medicine and General Practice as well as the trial center.

#### Plans for collection, laboratory evaluation, and storage of biological specimens for genetic or molecular analysis in this trial/future use {33}

Not applicable.

## Statistical methods

### Statistical methods for primary and secondary outcomes {20a}

Descriptive data will be used for all participant characteristics and scales as applicable (e.g., frequencies, means). Analyses of all scales will follow scale-specific recommendations. The confirmatory analysis for the primary endpoint is based on a generalized linear mixed models (GLMM) with a significance level of 5% (2-sided).

#### Primary endpoint

A GLMM is used because the primary endpoint is a patient-related outcome and these are embedded in the clusters. The model will include relevant patient covariates (e.g., age, gender). Taking the data’s cluster structure into account, the affiliation of patients to practice is included in the model as a random effect. The null hypothesis (no difference in BP control rate) will be rejected if the *p*-value for the Wald test statistics for the intervention effect is < 0.05. The *p*-value for the Wald test statistics for the intervention effect is < 0.05. The *p*-value for the Wald test statistics for the intervention effect is < 0.05. The adjusted odds ratio (OR) and the associated 95% confidence interval will be reported.

#### Secondary endpoints

All secondary analyses will be performed exploratively, i.e., without adjustment, using GLMM and adequate statistical standard procedures, taking into account the cluster structure of the data. A significance level of 5% will be assumed for all statistical analyses. Under individual randomization, an OR of 1.5 could be detected with 2 × 405 patients and a power of 80% and an OR of 1.6 with a power of 90% (Fisher’s exact test; PASS V14). We expect similar, probably slightly higher ORs for this CRT design.

#### Interim analyses {21b}

No interim analyses are planned.

## Methods for additional analyses (e.g., subgroup analyses) {20b}

Subgroup analyses will consider the age, gender, and socioeconomic status of the patients as well as practice and practice personnel characteristics.

### Methods in analysis to handle protocol non-adherence and any statistical methods to handle missing data {20c}

Robustness and sensitivity analyses with imputation procedures for the missing values will be performed.

#### Plans to give access to the full protocol, participant level-data, and statistical code {31c}

After study publication, the statistical code and trial data, including deidentified participant data, will be made available on request after approval of a formal written proposal. To gain access, researchers need to contact the corresponding author. This manuscript is the full study protocol, which is publicly available.

### Oversight and monitoring

#### Composition of the coordinating center and trial steering committee {5d}

The Institute of Family Medicine and General Practice is the coordinating center. The project management group consists of representatives from the coordinating center, the trial center, and the supporting statutory health insurances. A steering and review board with three national and international specialists is set up and will review all harms and reported adverse events.

#### Composition of the data monitoring committee, its role, and reporting structure {21a}

Data management and data monitoring are provided by the trial center (Center for Clinical Trials, University Hospital Essen, University of Duisburg-Essen, https://zkse.de/), which is independent from the sponsor and has no competing interests. All data-related procedures are carried out according to the standardized procedures defined in current SOPs. The data management system applies an integrated audit trail and is GCP compliant. All unexpected findings will be reported to the principal investigator (BW) who will decide upon the procedure together with the study’s advisory board.

#### Adverse event reporting and harms {22}

If adverse events or other unintended effects of the intervention occur during the course of the study, they will be documented, evaluated, and reported. All patients and physicians are asked for adverse events in the follow-up questionnaires. Throughout the study, safety analyses are performed for all patient-relevant endpoints. A steering and review board with three national and international specialists is set up and will review all harms and reported adverse events.

#### Frequency and plans for auditing trial conduct {23}

The documentation in the study folders is audited at baseline and follow-up by clinical monitors from the trial center responsible for data management and monitoring. This center is independent from the investigator and the sponsor.

#### Plans for communicating important protocol amendments to relevant parties (e.g., trial participants, ethical committees) {25}

In case of modifications to the protocol, the ethics’ committee and participants will be informed.

#### Dissemination plans {31a}

The results will be disseminated to participating practices, regional and national physician agencies and professional associations, statutory health insurances, patient representatives, and the scientific community and the public. Information channels will include websites, journal publications, conference presentations, newsletters to relevant stakeholders, and press releases. The study is supported by three statutory health insurances which will contribute to the dissemination.

## Discussion

The PIA-Intervention as a complex telemedicine intervention realizes an ICT-supported delegation model for German primary care. Involving physician-supervised medication adjustments by practice assistants, the project is a step towards more task delegation in German GP practices and advancement of practice assistants’ professional roles. Aiming at a high fit accuracy for GP practices, the development of the intervention applied three participatory strategies: (1) the project was initiated by a practicing, academic family medicine specialist; (2) agile software development with close interaction of medical software engineers, information system and implementation scientists, and an academic GP; and (3) repetitive testing and software adjustments involving practice personnel (GPs and practice assistants). Thus, it followed state-of-the-art principles of the implementation sciences [28].

During study conduct, several practical and operational issues evolved due to the SARS-CoV-2 pandemic requiring described protocol adjustment. First, there was a need for over-recruitment of practices due to unusual numbers of dropouts after the target number of practices had been successfully recruited initially. The consistently reported reasons were pandemic-related duties and strains. Given a limited overall project duration and the need for a prolonged recruitment period, the primary outcome was adjusted to a minimum follow-up of at least 6 months. This is justified by international studies of ICT-delegation models which showed significant improvements in hypertension control already after this shorter period [9, 16, 17]. Second, patient recruitment by practices is more difficult as patients visit the practices less frequently given recommendations for social distancing and lock-down regulations. Therefore, the targeted power was adjusted from 0.9 to 0.8 leading to a reduced target sample size. Third, there was a higher need for individual support of practice teams by the research team due to the pandemic, e.g., active support for patient recruitment and telephone reminders for motivation.

The project is supported by the Federal Joint Committee (G-BA) within a legal framework allowing for special contracting option (so-called selective contracts) according to the German social security code V (SGB V, §75a Selektivvertraege). This implies not only a scientific evaluation of new care models but also a preparation for potential implementation in routine care by special contracts which regulate health services (here PIA-ICT) including reimbursement. Thus, if proven effective, the PIA-ICT will be considered for the benefit catalog of the statutory health insurance funds (GKV) by the Federal Joint Committee (G-BA).

## Trial status

Recruitment started on May 1, 2020, and is scheduled to be completed by March 31, 2021.

## Supplementary Information

**Additional file 1: Table S2.** Overall schedule of enrolment, intervention, assessments, and time commitment for trial participants. Is an overview for overall schedule of enrolment, intervention, assessments, and time commitment for patients, general practitioners and practice assistants.

## Abbreviations

AES: Advanced Encryption Standard
BP: Blood pressure
DBP: Diastolic blood pressure
GCP: Good Clinical Practice
GLMM: Generalized linear mixed models
GP: General practitioner
ICT: Information Communication Technology
MARS-D: Medication Adherence Rating Scale – Deutsch
OR: Odds ratio
PIA: PC-gestütztes Fallmanagement von Hypertonikern zur Implementierung einer Leitlinien-konformen Hypertonietherapie anhand eines Arzt-definierten und – supervidierten, patientenindividuellen Therapiealgorithmus (= official German title which stands for a “PC supported case management of hypertensive patients to implement guideline-based hypertension therapy using a physician-defined and -supervised, patient-specific therapeutic algorithm”)
SBP: Systolic blood pressure
SOP: Standard operating procedure
SUS: System Usability Scale
TLS: Transport Layer Security
UTAUT: Unified Theory of Acceptance and Use of Technology
WHO: World Health Organization
WHO5: World Health Organization-Five Well-Being Index
