# Supplementary figures and images for: Optimizing blood pressure control by an Information Communication Technology-supported case management (PIA study): study protocol for a cluster-randomized controlled trial of a delegation model for general practices
Source: Trials. 2021 Oct 25;22:738. doi: 10.1186/s13063-021-05660-4 (PMC8543417; doi:10.1186/s13063-021-05660-4)

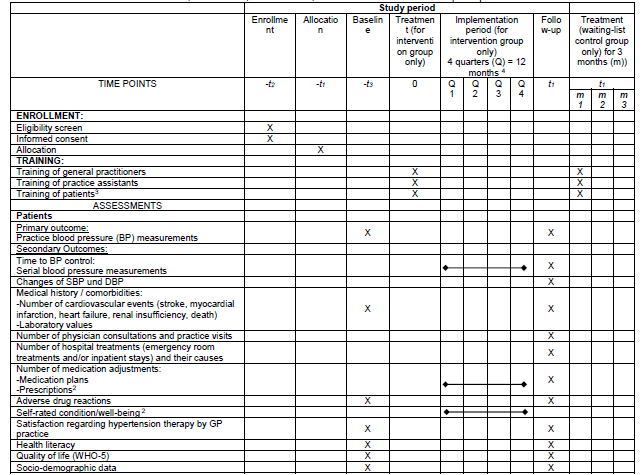

Supplement: Supplementary file 1 — Additional file 1: Table S2. Overall schedule of enrolment, intervention, assessments, and time commitment for trial participants. Is an overview for overall schedule of enrolment, intervention, assessments, and time commitment for patients, general practitioners and practice assistants. [file 13063_2021_5660_MOESM1_ESM.zip › ADDfile 1.PNG]

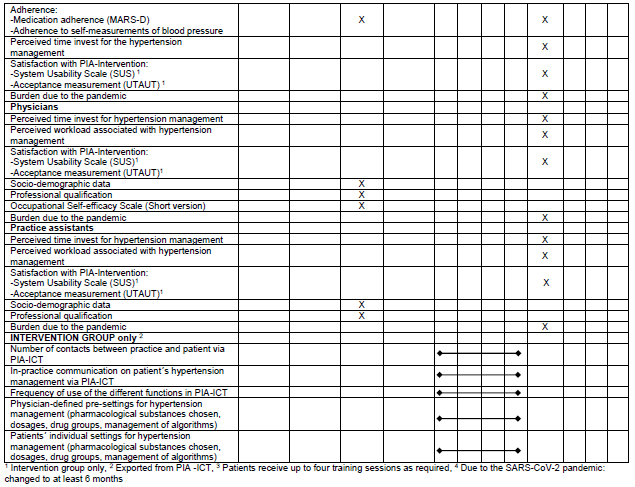

Supplement: Supplementary file 1 — Additional file 1: Table S2. Overall schedule of enrolment, intervention, assessments, and time commitment for trial participants. Is an overview for overall schedule of enrolment, intervention, assessments, and time commitment for patients, general practitioners and practice assistants. [file 13063_2021_5660_MOESM1_ESM.zip › ADDfile 2.PNG]
